# Increased levels of 3-hydroxykynurenine parallel disease severity in human acute pancreatitis

**DOI:** 10.1038/srep33951

**Published:** 2016-09-27

**Authors:** Christos Skouras, Xiaozhong Zheng, Margaret Binnie, Natalie Z. M. Homer, Toby B. J. Murray, Darren Robertson, Lesley Briody, Finny Paterson, Heather Spence, Lisa Derr, Alastair J. Hayes, Andreas Tsoumanis, Dawn Lyster, Rowan W. Parks, O. James Garden, John P. Iredale, Iain J. Uings, John Liddle, Wayne L. Wright, George Dukes, Scott P. Webster, Damian J. Mole

**Affiliations:** 1 Clinical Surgery, The University of Edinburgh, United Kingdom; 2 Medical Research Council, Centre for Inflammation Research, Queen’s Medical Research Institute, The University of Edinburgh, United Kingdom; 3 University/British Heart Foundation Centre for Cardiovascular Science, The University of Edinburgh, United Kingdom; 4 Mass Spectrometry Core, Edinburgh Clinical Research Facility, The University of Edinburgh, United Kingdom; 5 GlaxoSmithKline, Stevenage, United Kingdom; 6 Wellcome Trust Clinical Research Facility, The University of Edinburgh, United Kingdom; 7 National Technical University of Athens, Greece; 8 Edinburgh and Lothians Laboratory Medicine, United Kingdom; 9 Discovery Partnerships with Academia, GlaxoSmithKline, Stevenage, United Kingdom; 10 Bioanalysis, Immunogenicity and Biomarkers, GlaxoSmithKline, Ware, United Kingdom; 11 Academic Discovery Performance Unit, Alternative Discovery & Development, GSK, Raleigh, North Carolina, United States of America

## Abstract

Inhibition of kynurenine 3-monooxygenase (KMO) protects against multiple organ dysfunction (MODS) in experimental acute pancreatitis (AP). We aimed to precisely define the kynurenine pathway activation in relation to AP and AP-MODS in humans, by carrying out a prospective observational study of all persons presenting with a potential diagnosis of AP for 90 days. We sampled peripheral venous blood at 0, 3, 6, 12, 24, 48, 72 and 168 hours post-recruitment. We measured tryptophan metabolite concentrations and analysed these in the context of clinical data and disease severity indices, cytokine profiles and C-reactive protein (CRP) concentrations. 79 individuals were recruited (median age: 59.6 years; 47 males, 59.5%). 57 met the revised Atlanta definition of AP: 25 had mild, 23 moderate, and 9 severe AP. Plasma 3-hydroxykynurenine concentrations correlated with contemporaneous APACHE II scores (R^2^ = 0.273; Spearman rho = 0.581; P < 0.001) and CRP (R^2^ = 0.132; Spearman rho = 0.455, P < 0.001). Temporal profiling showed early tryptophan depletion and contemporaneous 3-hydroxykynurenine elevation. Furthermore, plasma concentrations of 3-hydroxykynurenine paralleled systemic inflammation and AP severity. These findings support the rationale for investigating early intervention with a KMO inhibitor, with the aim of reducing the incidence and severity of AP-associated organ dysfunction.

Acute pancreatitis (AP) is a sterile, localized inflammation of the pancreas. In approximately 25% of affected patients, AP results in a systemic inflammatory response leading to multiple organ dysfunction syndrome (MODS)^1^,^2^. MODS commonly develops early after the onset of AP^3^. Persons who develop AP–MODS require invasive monitoring and organ support in a critical care environment, with an in-hospital mortality rate that may reach 36–50%^4^,^5^. Moreover, survivors of AP-MODS require a prolonged hospital stay and have a reduced overall life expectancy compared to those with mild AP^6^. Therefore, early recognition and stratification of AP severity is essential.

The exact pathological mechanisms that drive AP–MODS remain unclear, but the kynurenine pathway of tryptophan metabolism (Fig. 1) is emerging as a potentially important contributory mechanism^7^,^8^. In an inflammatory context, metabolism of tryptophan through the kynurenine pathway is dramatically increased through induction of indoleamine 2,3-dioxygenase (IDO) expression by pro-inflammatory cytokines, in particular, interferon gamma (IFN-γ)^9^. IDO upregulation increases substrate flux through downstream enzymes, in particular kynurenine 3-monooxygenase (KMO), thereby generating biologically–active metabolites that participate in multiple physiological and pathological processes^10^,^11^,^12^,^13^,^14^,^15^. In rodent models of AP, 3-hydroxykynurenine, the product of the gate-keeper enzyme kynurenine 3-monooxygenase (KMO), appears to be critical to the pathogenesis of AP-MODS^7^. 3-hydroxykynurenine causes tissue injury via oxidative stress and pathological cross-linking of proteins^16^. Therapeutic blockade of KMO by genetic deletion in mice or pharmacological inhibition in rats reduces 3-hydroxykynurenine formation and protects against lung and kidney injury in experimental models of AP^7^. Other metabolites in the kynurenine pathway have been postulated to mediate immunological tolerance dependent on signalling via the aryl hydrocarbon receptor, whilst kynurenine specifically may contribute to T-helper Th17 and T-regulatory lymphocyte differentiation^17^,^18^.

In critically ill individuals, rapid and profound tryptophan degradation can occur^19^. Indeed, tryptophan depletion and/or elevated concentrations of kynurenine metabolites have been detected in the plasma of major trauma patients, and in patients following cardiac bypass surgery, while elevated serum kynurenine concentrations are associated with the need for renal replacement therapy and invasive ventilation in persons with AP-MODS^8^,^11^,^20^,^21^. However, the detailed profile of the kynurenine pathway and its role in the pathophysiology of AP has not been elucidated. In considering the therapeutic potential of KMO inhibition for AP, it is important to establish the precise timing and magnitude of activation of the kynurenine pathway in relation to the onset of AP and the development of AP-MODS, the inflammatory burden and overall AP severity. The aim of the present study, therefore, was to define the precise temporal and quantitative relationship between kynurenine pathway metabolism and the onset and severity of AP-associated organ dysfunction.

## Results

### Recruitment Performance

**Figure 2:**
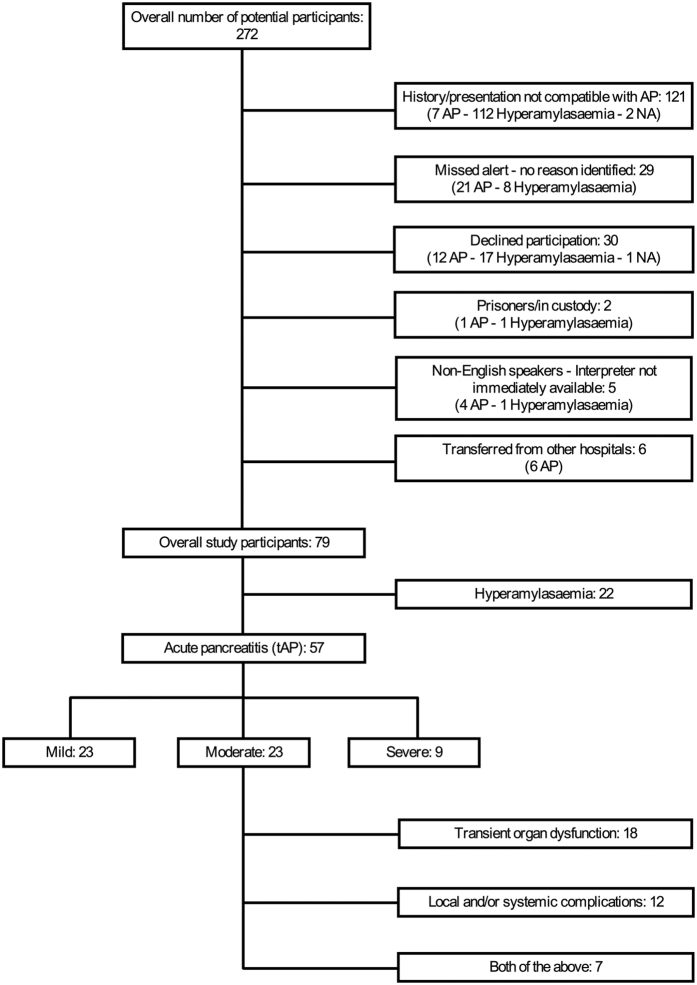
CONSORT diagram of the IMOFAP study. Reasons for patient exclusion are summarised, with post-hoc diagnoses for each exclusion category included in brackets (NA = not available). Patients of the moderate AP severity group were divided to three further sub-groups according to the revised Atlanta guidelines for explanatory purposes.

**Figure 3:**
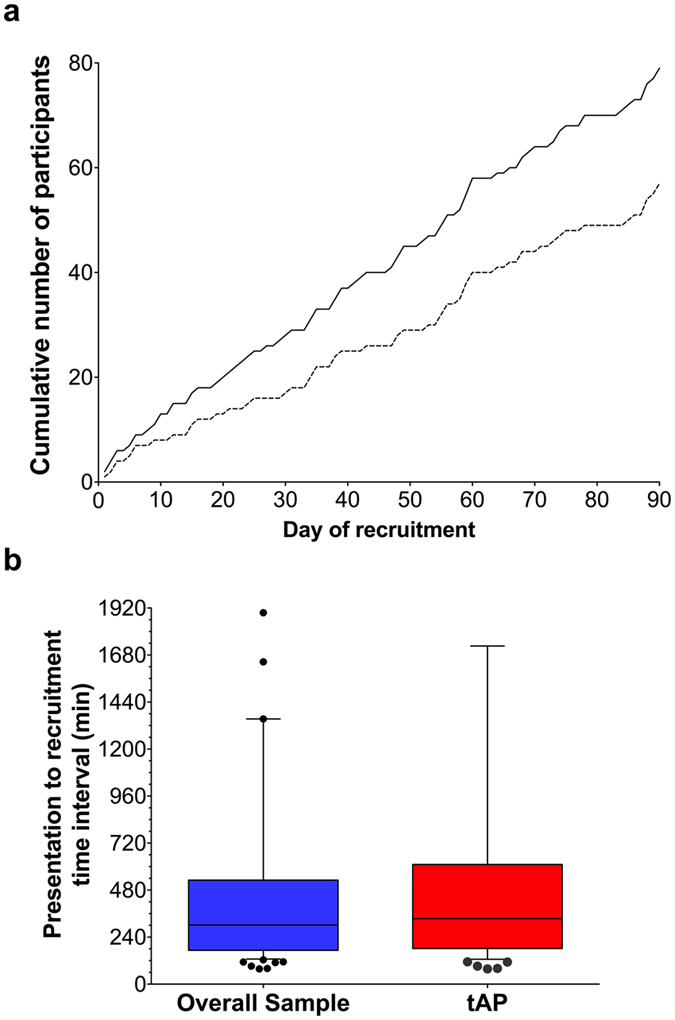
(**a**) Cumulative number of enrolled participants per calendar day of the recruitment phase of the study. Solid line: overall study participants; dashed line; tAP participants. (**b**) Box & whiskers plot of the time interval from patient presentation to hospital to study recruitment, in minutes. Blue box: overall study participants; red box tAP participants. Boxes: 1^st^ and 3^rd^ quartile; horizontal lines: median values; error bars:1^st^ and 9^th^ deciles; dots: outlying values beyond 1^st^ and 9^th^ decile.

A total of 79 patients were recruited, 57 patients (72.2%) of whom were diagnosed with AP in accordance with the revised Atlanta guidelines (“true AP” – tAP). Of the 57 tAP patients, 10 had an amylase level below 300 upon presentation, and either a subsequent amylase rise or a confirmation of AP by imaging. The demographic characteristics of the study participants are summarized in Supplementary Table S1, and a CONSORT diagram is shown in Fig. 2. There was no effect of day of the week (P = 0.317) or time of day (P = 0.397) on recruitment. Moreover, time of day for recruitment (T0) followed a random distribution. The cumulative frequency of participant enrolment by calendar day of the recruitment phase and the relative frequency of enrolment by weekday are shown in Fig. 3a and Supplementary Table S2, respectively. No systematic bias was present for the twenty-nine potentially eligible patients that were not recruited. Thirty patients declined to participate, of whom 12 had AP and 3 required admission to critical care. One male participant formally withdrew from the study within 12 hours from recruitment.

Sampling performance was efficient, and a total of 508 samples were obtained and analysed. Due to the high temporal resolution design of the study, some sample time-points were missed, but no systematic bias was identified for the non-obtained samples. The median time from acute presentation to hospital (pre-recruitment sample) to recruitment was 5 hours 34 minutes (IQR: 182–611 minutes) for tAP participants (Fig. 3b). This interval included examination and consultation by the direct clinical care team(s), routinely collected acute presentation sample (pre-recruitment) receipt, processing and analysis, verification of the result by the hospital laboratory staff and contact with the study team for verification of the clinical history, and informed consent.

### Inflammatory Indices, Composite Scores & Cytokines

**Figure 4:**
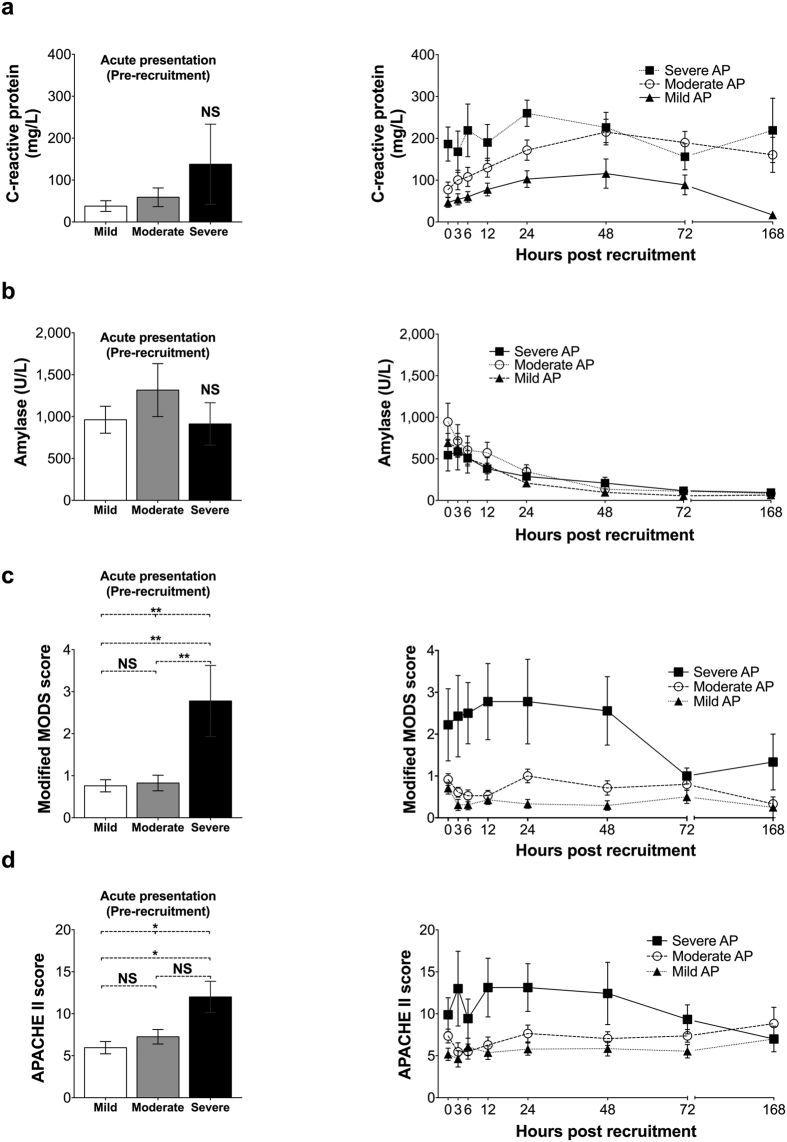
Panel of comparisons between mild (n = 25), moderate (n = 23) and severe AP (n = 9) participant groups for (**a**) C-reactive protein; (**b)** Amylase; (**c**) Modified MODS score (as described in the revised Atlanta guidelines); (**d**) APACHE II score. Bar charts on the left hand side of the panel represent Tminus sample means (acute presentation – pre-recruitment), while right-hand side plots depict the time-course for each variable mean per participant group. NS: not statistically significant difference; *0.05 > P > 0.01; **P < 0.01; error bars represent standard errors of the mean.

A clear rise in CRP level was evident for tAP participants (standardized AUC for CRP concentration, median (IQR): hyperamylasaemia participants: 18.9 (5.75–45.4) mg/L; tAP participants: 126.0 (49.9–201.1); P < 0.001), the magnitude of which was proportionate to AP severity (Fig. 4a) and in keeping with a substantial systemic inflammatory insult (standardized AUC for CRP concentration, median (IQR): mild: 58.9 (26.3–131.2) mg/L; moderate: 148.1 (74.5–243.9) mg/L; severe: 225.6 (174.9–260.2) mg/L; P = 0.001). The acute presentation (pre-recruitment) mean CRP concentrations were higher in severe AP, but this difference was not statistically significant (CRP concentration, median (IQR): mild: 11 (5–34) mg/L; moderate: 19 (9–39) mg/L; severe: 59 (30–65) mg/L; P = 0.166) (Fig. 4a and Supplementary Table S3). Resolution of inflammation, indicated by a fall in CRP, occurred between 48 and 72 hours for the majority of patients. Conversely, serum amylase was more than three times higher than the upper limit of the normal range upon recruitment, and normalised within 24 hours for the majority of participants. There was no association between serum amylase level upon presentation and disease severity (Fig. 4b). Furthermore, mean and minimum concentrations of albumin, as well as the corresponding mean standardized AUC for albumin were significantly lower in the severe AP group (Supplementary Table S3).

Severe AP patients had a significantly greater modified MODS score at presentation, when compared to the other two participant groups (mean MODS score, (SD; range): mild: 0.8 (0.7; 0–3); moderate: 0.8 (0.9, 0–4); severe: 2.8 (2.5; 0–8); P = 0.003), followed by a further rise during the initial 12-hour period after recruitment and apparent resolution (a reduction in MODS score to ≤2) beyond the 48-hour mark (Fig. 4c). No association was discovered between the time interval from the onset of symptoms to recruitment and the modified MODS score upon presentation of severe AP patients (Spearman’s rho = −0.037, P = 0.926). Both day 1 APACHE II and T_minus_ APACHE II scores were higher in the severe AP group (day 1 APACHE II score, mean (SD): mild: 8.2 (4.4); moderate: 11.4 (3.8); severe 19.3 (11.0); P = 0.001, and T_minus_ APACHE II score, mean (SD): mild: 6.0 (3.6); moderate: 7.6 (3.8); severe: 12.0 (5.6); P = 0.002). APACHE II scores also declined after 48 hours, with the decrease being more prominent from T72 onwards (Fig. 4d). Importantly, the decline of both modified MODS and APACHE II scores (calculated for each time-point) coincided with and is due to the deaths of two patients in the severe AP group, who died within 55 and 101 hours after recruitment. The respective scores for the surviving severe AP patients remained relatively unchanged for the duration of their participation in the study.

The inflammatory burden of the study cohort was further quantified by supplementary serial measurements of pro- and anti-inflammatory cytokines over the same time-points. The severe AP patient group was found to have significantly higher levels of peak and mean concentrations, as well as standardized AUC levels of IL-1B, IL-6, IL-10, TFF3, and CD163 (Supplementary Table S3).

### Tryptophan Metabolites

Summary measures for the concentration of tryptophan and all studied tryptophan metabolites are presented in Table 1. Tryptophan depletion was marked and proportionate to disease severity and especially profound in severe AP (standardised AUC for tryptophan concentration, mean (SD): mild: 24423.9 (8803.9) ng/mL; moderate: 19721.4 (8140.5) ng/mL; severe: 13782.4 (3789.8) ng/mL; P = 0.004). Importantly, upon presentation to hospital, no statistically significant difference in tryptophan concentration levels was evident among the three groups (tryptophan concentration, mean (SD): mild 29796.6 (10939.7) ng/mL; moderate: 26133.0 (9973.8) ng/mL; severe: 24273.7 (9926.4) ng/mL; P = 0.281). Moreover, the observed effect on tryptophan levels was not a result of dilution due to resuscitation, as no statistically significant difference was observed in the standardised AUC of haematocrit^22^ between the three groups (standardized AUC for haematocrit, mean (SD): mild 0.361 (0.04); moderate: 0.350 (0.045); severe 0.330 (0.034); P = 0.161).

**Figure 5:**
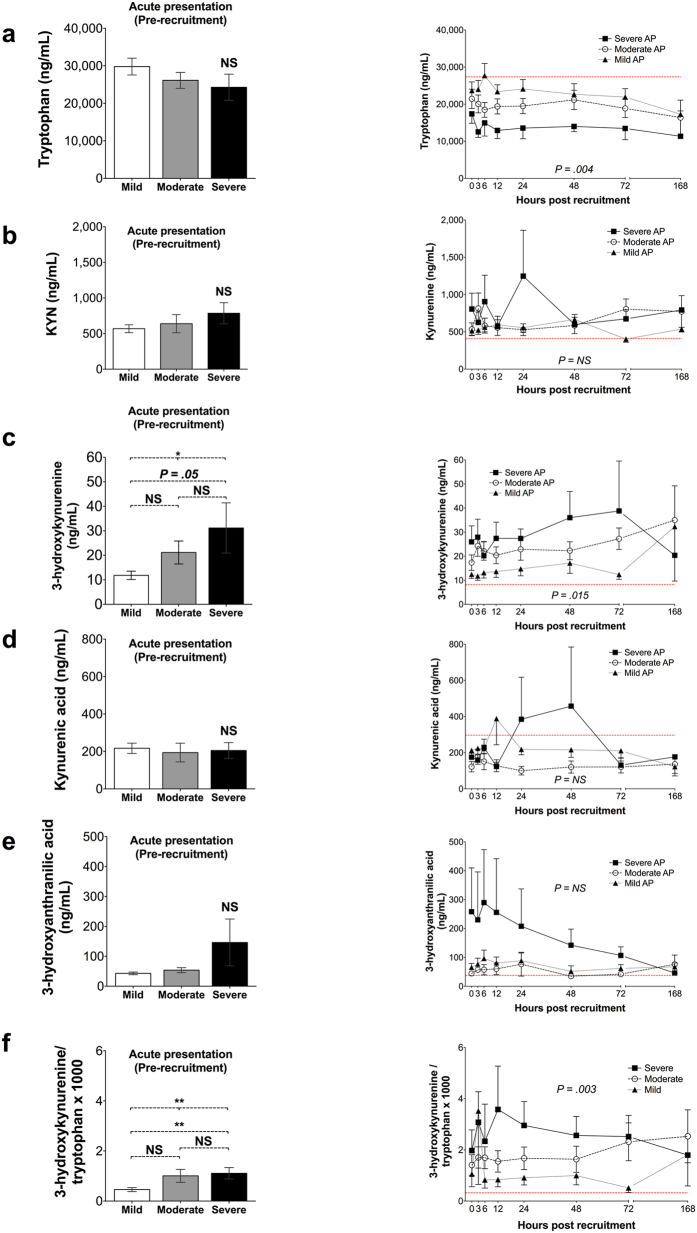
Plasma concentrations of tryptophan and the kynurenine pathway metabolites in tAP participants over time, grouped by AP severity according to the revised Atlanta criteria (group sizes at recruitment: mild n = 25; moderate n = 23; severe n = 9 individuals). (**a**) Tryptophan; (**b**) Kynurenine; (**c**) 3-hydroxykynurenine; (**d**) Kynurenic acid; (**e**) 3-hydroxyanthranilic acid; (**f**) 3-hydroxykynurenine to tryptophan ratio multiplied by 1000. For all panels, data points represent means and error bars represent standard errors of the mean. P-values for between-group comparisons of standardised AUC are appended. NS: not statistically significant. Dashed red lines represent healthy volunteer (n = 8) mean concentration of each metabolite.

No significant differences in peak level, mean level or standardized AUC of kynurenine, kynurenic acid, and 3-hydroxyanthranilic acid were detected among the three patient groups (Table 1). In specific, the observed difference of 3-hydroxyanthranilic acid concentration plots on Fig. 5e, although visually substantial, did not reflect a statistically significant difference, and was driven by the extreme values on two individual participants in the severe AP group.

The severe AP patient group had a significantly higher peak 3-hydroxykynurenine concentration across all study time-points (peak 3-hydroxykynurenine concentration, median (IQR): mild: 13.5 (8.6–17.8) ng/mL; moderate: 20.0 (12.1–46.6) ng/mL; severe: 25.2 (14.3–35.6) ng/mL; P = 0.013). The standardized AUC for 3-hydroxykynurenine was also significantly different among the three groups, although only a marginal difference was observed between the moderate and the severe group, as shown on Table 1 (standardized AUC for 3-hydroxykynurenine concentration, median (IQR): mild: 10.81 (7.4–13.9) ng/mL; moderate: 18.2 (11.4–38.1) ng/mL; severe: 21.6 (11.7–28.0) ng/mL; P = 0.015). The median time interval required to reach peak 3-hydroxykynurenine concentration was 24 hours (95% C.I.: 0.0–49.0 hours) in the severe AP group, compared to 12.0 hours (95% C.I.: 6.7–17.3 hours) in the mild AP group and to a mean time interval of 46.0 hours (95% C.I.: 32.9–59.0 hours) in the moderate group (P = 0.019).

To provide an indication of flux through KMO we calculated the 3-hydroxykynurenine/tryptophan ratio, multiplied by 1000 for practical purposes. We observed a significant increase in KMO flux in proportion to disease severity (standardized AUC for 3-hydroxykynurenine/tryptophan ratio x 1000, median (IQR): mild: 0.45 (0.26–0.89); moderate: 0.73 (0.51–2.64); severe: 1.67 (0.93–1.84); P = 0.003). Time-plots for the concentration of each analysed metabolite are depicted on Fig. 5.

**Figure 6:**
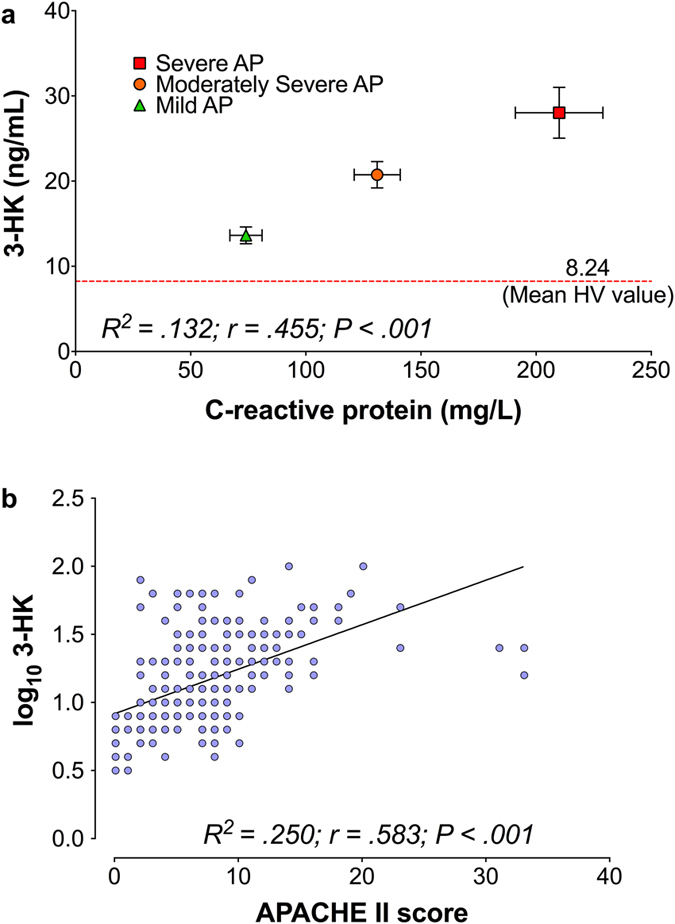
Dot-plots of correlations between (**a**) Plasma concentrations of C-reactive protein and 3-hydroxykynurenine, for the tAP cohort grouped by AP severity (mild AP n = 25; moderate AP n = 23; severe AP n = 9). Data points represent means; error bars show standard deviation on both axes. The dashed red line represents mean 3-hydroxykynurenine plasma concentration in healthy volunteers (n = 8). (**b**) APACHE II score and contemporaneous log_10_ 3-hydroxykynurenine plasma concentrations. Due to the non-normal distribution of plasma concentrations of of 3-hydroxykynurenine, a logarithmic transformation was used for the correlation to provide a better fit. For both correlations, results from samples obtained from T0 up to and including T48 were analysed. Results of respective Spearman correlations have been appended on each figure of the panel.

When considering all samples obtained from T0 up to and including T48, logarithmic 3-hydroxykynurenine levels correlated well with contemporaneous CRP (R^2^ = 0.132; rho = 0.455, P < 0.001) (Fig. 6a) and APACHE II score (R^2^ = 0.250; rho = 0.583; P < 0.001) (Fig. 6b). Furthermore, moderate correlation of logarithmic 3-hydroxykynurenine was discovered with contemporaneous levels of TFF3 (rho = 0.604, P < 0.001), albumin (rho = −0.568, P < 0.001), creatinine (rho = 0.531, P < 0.001), TNF-a (rho = 0.462, P < 0.001), and RAGE (rho = 0.455, P < 0.001), and weaker correlation with IL-10 (rho = 0.357, P < 0.001), IL-6 (rho = 0.352, P < 0.001), IL-17a (rho = 0.349, P < 0.001), CD163 (rho = 0.298, P < 0.001), IL-8 (rho = 0.293, P < 0.001), TNFS10 (rho = −0.259, P < 0.001), chemerin (rho = 0.247, P < 0.001), IL-1 (rho = 0.220, P < 0.001), insulin C-peptide (rho = 0.230, P < 0.001), cardiac troponin I (rho = 0.217, P < 0.001), CXCL12 (rho = 0.195, P = 0.001), and CD40 ligand (rho = 0.181, P = 0.003). Conversely, no correlation was found with insulin (rho = 0.057, P = 0.352), CA 15-3 (rho = −0.079, P = 0.199), B7-H1 (rho = −0.062, P = 0.312), and IFN-γ (rho = −0.019, P = 0.758). Differences between participant groups for all studied cytokines are summarized in Supplementary Table S3, and post-hoc pairwise comparisons of significantly different standardized AUC are depicted on Supplementary Table 4.

## Discussion

The kynurenine pathway of tryptophan metabolism and especially KMO, the enzyme that determines the metabolic fate of kynurenine, is increasingly recognized as a key contributor to the pathogenesis of AP-MODS. The present study precisely defines the temporal profile of kynurenine pathway metabolite concentrations in peripheral blood in relation to the onset and severity of AP in humans. Our data show a noteworthy association between plasma concentrations of 3-hydroxykynurenine and AP severity as defined by standard classification systems. Furthermore, an important correlation was discovered between 3-hydroxykynurenine concentrations and the systemic inflammatory burden measured by CRP levels. Additionally, we observed the classical paradigm pro-inflammatory cytokine profile in AP, namely TNF-α and IL-6, as well as RAGE and TFF3, that correlated strongly with 3-hydroxykynurenine concentrations in this cohort. Our novel findings add depth to the previously reported association between serum kynurenine concentrations and the requirement for invasive renal and respiratory support during AP-MODS^8^. Moreover, these findings build on our recent discovery that genetic deletion of KMO in mice and administration of a highly-specific KMO inhibitor protects against lung and kidney injury in experimental rodent models of AP^7^. Together, these data strongly support the translational potential of KMO inhibition as a therapeutic strategy to protect against MODS in human AP.

Strengths of this study include the efficiency of recruitment, sampling process, sampling frequency and high coverage, thus minimal sampling bias. Based on the knowledge that AP and the consequent systemic inflammatory response evolve rapidly, we hypothesise that KMO inhibition is likely to be most effective when delivered as early as possible in the course of the disease and therefore sought to define the evolving trajectory of the inflammatory response with high definition during the early phase.

The burden of systemic inflammation in the cohort as measured by the magnitude of CRP rise was clear. The fact that APACHE II score correlated well with disease severity as classified by the revised Atlanta criteria, and that the modified MODS score followed the same trajectory as CRP, provide additional reassurance that interpreting plasma concentrations of 3-hydroxykynurenine and other kynurenine metabolites in this context is valid and appropriate.

In the present study, the magnitude of tryptophan depletion was proportional to AP severity and was more prominent over time, suggesting increased tryptophan metabolism. Although tryptophan levels can fluctuate diurnally^23^,^24^, diurnal variation does not account for the difference in tryptophan metabolism between AP severity strata, because the time of day at which recruitment occurred was distributed randomly. Furthermore, although it is possible that fasting due to hospital admission and/or critical illness may in theory account for part of the observed tryptophan depletion, Poesen *et al*.^25^ previously demonstrated that plasma levels of tryptophan, kynurenine, and kynurenic acid were not significantly different in healthy humans with low or high protein intake, and one would not expect to observe a correlation with AP severity if fasting was the cause. The extent of tryptophan depletion is too great to be due to haemodilution after intravenous fluid resuscitation, and there was no observed change in the haematocrit or dilution effect seen in other plasma analytes. It therefore seems reasonable to conclude that the observed decrease in tryptophan levels is likely to be the result of increased metabolism.

The increase in steady state 3-hydroxykynurenine concentrations in plasma is consistent with increased flux through the kynurenine pathway. This change is proportionate to disease severity and augments with time. Importantly, elevated 3-hydroxykynurenine is associated with increased inflammatory burden, as indicated by the correlation of plasma 3-hydroxykynurenine levels with contemporaneous CRP concentrations. While it is curious that no significant changes were observed in other tryptophan metabolites, the indications from studies in rodents are that KMO represents the predominant route for this pathway. For this reason, the ratio between 3-hydroxykynurenine to tryptophan (multiplied by 1000 for practical purposes) was used to try and express the pathway flux through KMO. This ratio was found to be time-dependent and proportionate to AP severity, with a lead-time of 12 hours when compared to the timing of the peak serum CRP that occurred at 24 hours for the severe AP group. Lastly, the elevation in 3-hydroxykynurenine/tryptophan ratio was greater in those few patients with specific respiratory, renal and cardiac dysfunction as defined by those specific components of the MODS score.

In conclusion, this study demonstrates that metabolic flux through KMO is elevated proportionately to disease severity in human AP. Plasma concentrations of 3-hydroxykynurenine correlate with the burden of inflammation, incidence of organ dysfunction and AP severity. These findings reinforce the rationale for investigating early phase KMO inhibition as a therapeutic strategy to protect against AP-MODS in human AP.

## Methods

### Ethics & Regulatory Approvals

This study was approved by the Scotland A Research Ethics Committee (REC) (REC reference number: 13/SS/0136, amended REC REF AM01), and NHS Lothian Research & Development committee (Project Number: 2013/0098 and SA1). The study sponsor was the Academic and Clinical Central Office for Research and Development (ACCORD), a collaboration between the University of Edinburgh and NHS Lothian. Permission to access confidential medical records was granted by the NHS Lothian Caldicott Guardian. Written informed consent was obtained from all participants or their legal representative. Prior to commencing the study, a summary of the study protocol was registered in the public domain on the UK Clinical Trials Gateway (former UK Clinical Research Network) (registration number: 16116)^26^. The study was conducted in accordance with the University of Edinburgh and NHS Lothian guidance. Adults without the capacity to give informed consent were recruited in accordance with the Adults with Incapacity (Scotland) Act 2000, Part 5^27^. Informed consent was sought when capacity was regained.

### Research Sites

Recruitment of participants was undertaken in the Emergency Department (ED), and General Surgery wards at the Royal Infirmary of Edinburgh (RIE). The Wellcome Trust Clinical Research Facility (WTCRF) provided research nurse support for recruitment and consent, data collection, sample handling and storage.

### Inclusion and Exclusion Criteria

Any patient over the age of 16 years with a potential or confirmed new diagnosis of AP was identified as a potential participant. For the diagnosis of potential AP, two of the following three features were required:

1. A clinical history of symptoms compatible with AP (i.e. abdominal pain, nausea, and/or vomiting);
2. Serum amylase concentration greater than the upper limit of the reference range (>100 IU/L). This threshold was decided upon in order to capture potential participants with an amylase level below the threshold of the revised Atlanta definition for AP, due to a late or atypical presentation. A serum amylase concentration in excess of 300 IU/L was required as a feature for the diagnosis of true AP (tAP).
3.  Evidence of AP on computerised tomography (CT) and/or abdominal ultrasound scan (AUSS).

Persons under the age of 16 years, prisoners and persons in police custody were excluded from the study.

### Recruitment

In order to capture potential participants as early as possible in the course of the disease, an automatic alert was initiated from the biochemistry laboratory for any serum sample with a measured elevated amylase concentration and transmitted to the WTCRF research nurses on a dedicated study mobile telephone. The dedicated study telephone number was also distributed to all ED and surgical team members. Upon each alert, the investigator or WTCRF nurse checked the clinical history of each potential study participant via his or her electronic records on InterSystems TrakCare^**®**^. If the clinical presentation was compatible with AP, the patient was visited and suitability for recruitment was confirmed. A member of the direct clinical care team made the initial approach to each potential participant, prior to informed consent and recruitment by the WTCRF nurse team.

The recruitment phase lasted for 90 calendar days – from 17^th^ September 2013 to 16^th^ December 2013 – and was carried out 24 hours per day, 7 days per week with support from 30 WTCRF research nurses on a 12-hour shift rota supported by 2 clinical support workers and a clinical technician.

### Healthy Volunteers

Healthy volunteers over the age of 18 years were recruited with ethical approval (REC reference number: 08/S1103/38, United Kingdom). Volunteers were excluded from participation on the basis of the presence of any of the following: renal dysfunction (eGFR < 30 mL/min); hepatic dysfunction (Child-Pugh score B or C); pregnancy or breast feeding; blood dyscrasia or anaemia (haemoglobin <12 g/dL); active malignancy; chronic inflammatory condition; any intercurrent illness; and any recent surgical procedure.

### Data Collection and Data Management

Data were collected from individual patient charts, clinical case notes and the following electronic sources:

1. WardWatcher software (Scottish Intensive Care Society Audit Group - SICSAG).
2. TrakCare Patient Management System (InterSystems, Massachusetts, USA).
3. SCI-Store data repository (SCI NHS National Services Scotland).
4. Picture Archiving and Communication System (PACS).
5. Emergency Care Summary (ECS) database (National Information Systems Group).
6. iLaboratory information system (iLab or APEX).

Data were entered into a dedicated, password-protected and software-encrypted study laptop with regular back-up. Participant data were link-anonymized and securely stored separately from the identifiers in accordance with the secure data protection principles of the sponsor and the WTCRF. A bespoke data collection template was created in Java™ with set limits to avoid the potential for mistyping data entry and mandatory completion to ensure comprehensive recording. Input from the data collection software was made automatically to a custom Microsoft^®^ Access^®^ study database. A diarized alert/reminder system was activated on initiation of a new patient at the time of recruitment, that sent email and screen notifications to the study nurses to ensure time points were not missed. This was especially important in cases of simultaneously enrolled and overlapping participants to ensure all time points were met (Supplementary Figure S5). In addition, WTCRF study nurses kept a hand-written log to enter free text, notes and other information pertinent to the study for which there was no appropriate data field in the electronic study database. Cross-referencing with the study database was performed at intervals.

### Sampling and Sample Handling

Peripheral blood was sampled at recruitment (T0) and 3, 6, 12, 24, 48, 72 and 168 hours (7 days) afterwards. In addition, the serum sample obtained at presentation to hospital (pre-recruitment, T_minus_) was retained by the Biochemistry Department and subsequently retrieved by WTCRF staff. Blood was sampled into gel clot-activator tubes for serum and into EDTA tubes for plasma and subsequently centrifuged at 3200 rpm for 8–10 minutes. All centrifuges were serviced and calibrated prior to the start of the study. Serum and plasma were aliquoted and immediately frozen at −80 °C until transfer on dry ice to long-term storage in a monitored, dedicated −80 °C freezer. A sample history and tracking log was kept for all events. There were no breaches of the sample handling/storage protocol. Additionally, routine haematological and biochemical analyses were performed in accordance with standard hospital protocols used in clinical care. Serum C-reactive protein (CRP) was used to define the inflammatory burden of the cohort.

### Composite Clinical Scores

For each participant, the APACHE II score was calculated on the day of admission, by using the most extreme values of the variables of interest (day 1 APACHE II) and on per time-point basis^28^. Similarly, the Marshall multiple organ dysfunction syndrome (MODS) score, modified as per the revised Atlanta Classification of AP, was calculated for each patient at each time-point and on a daily basis^4^,^29^.

### Tryptophan Metabolite Measurement

Kynurenine pathway metabolites were measured in plasma by two separate liquid chromatography–tandem mass spectrometry (LC-MS/MS) methods. For the detection of tryptophan, kynurenine, kynurenic acid and 3-hydroxyantranilic acid, plasma (5 μl) was initially diluted in phosphate-buffered saline (45 μl). The diluted plasma was added to 5 mM ammonium formate containing 0.1% trifluoroacetic acid (130 μl). Protein was precipitated by the addition of ice-cold 100% trichloroacetic acid (20 μl), samples were vortexed briefly, incubated for 30 minutes at 4 °C and centrifuged to obtain the supernatant. Separate standard curves were used to quantify levels of the individual metabolites. For tryptophan, the standard curve consisted of serial dilutions of d5-tryptophan (as surrogate analyte of tryptophan) in 10% pooled human plasma. For kynurenine, 1% BSA in phosphate-buffered saline was used. For kynurenic acid and 3-hydroxyanthranilic acid, 0.2% BSA in phosphate-buffered saline was used. Samples (10 μl) were injected onto a Waters Select HSS XP column (3 mm × 100 mm, 2.5 μm, Waters, Elstree, Herts) using a Waters Acquity UHPLC system, coupled to an ABSciex QTRAP 5500 mass analyser. The flow rate was 0.35 mL/min at 25 °C. Separation was carried out using a water:methanol gradient (both containing 0.1% formic acid). Chromatographic conditions were 50% methanol rising to 60% over 60 seconds, then to 65% over 180 seconds; held for 110 seconds, returned to 50% over 10 seconds and re-equilibrated for a further 200 seconds, giving a total run time of 10.2 minutes. The mass spectrometer was operated in positive electrospray ionization mode. The multiple reaction monitoring (MRM) transitions for the protonated analytes were tryptophan (*m/z* 205-188), kynurenine (*m/z* 209-146), kynurenic acid (*m/z* 190-144) and 3-hydroxyanthranilic acid (*m/z* 154-136). The transitions for d5-tryptophan standard were (*m/z* 210-122). Collision energies were 11, 29, 31, 33 and 37 eV respectively.

For the analysis of 3-hydroxykynurenine, plasma (100 μl) was added to a 40:60 mixture of acetonitrile in water containing 0.1% formic acid (130 μl) and 50 ng/ml of internal standard. Protein was precipitated by the addition of trichloroacetic acid (33 μl), samples were vortexed briefly, incubated for 30 minutes at 4 °C and centrifuged to obtain the supernatant. A standard curve containing serial dilutions of 3-hydroxykynurenine in 10% BSA diluted with phosphate-buffered saline enriched with 50 ng/ml internal standard was used. 10 μL volumes of each sample were injected onto a Waters Select HSS XP column (30 mm × 100 mm, 2.5 μm, Waters Corp, Elstree, Herts) using a Waters Acquity UHPLC system, coupled to an ABSciex QTRAP 5500 mass analyser. The flow rate was 0.8 mL/min at 30 °C. Separation was carried out using a water:methanol gradient (both containing 0.1% formic acid). Conditions were 5% methanol rising to 95% over 150 seconds, returning to 5% over 30 seconds, giving a total run time of 4 minutes. The mass spectrometer was operated in positive ion electrospray mode. The transitions for the protonated analytes were 3-hydroxykynurenine (*m/z* 225-208) and internal standard (*m/z* 228-211). Collision energies were 20 and 12 eV respectively.

Data were acquired and processed using Analyst quantitation software 1.3 (ABI Sciex). Results from retrieved acute presentation (pre-recruitment) samples were excluded from the time-course analysis since serum-gel activator tube containers were routinely used by the clinical care teams, a fact that may have led to discrepancy upon comparison with results from the dedicated K3-Ethylenediaminetetraacetic acid containers that were used for the study.

Four samples obtained from two patients in the severe AP group returned values of kynurenine concentration that were greater than 20 standard deviations away from the remaining concentration values of the severe AP cohort, including temporally adjacent samples from the same patient. These samples were deemed to have resulted from contamination during sample analysis, were designated as extreme outliers and were removed from further analysis.

### Cytokine Analysis

Plasma cytokines were analysed using a custom-designed Human Magnetic Luminex Screening Assay according to the manufacturer’s instructions (R&D Systems, MN, USA). Levels of insulin, interleukin-1 beta (IL-1B), insulin C-peptide and IFN-γ were measured using kit LXSAHM-4, cancer antigen 15-3 (CA 15-3) using kit LXSAHM-1 and tumour necrosis factor alpha (TNFα), B7 homolog 1 (B7-H1), chemokine (C-X-C motif) ligand 12 (CXCL 12), cluster of differentiation 163 (CD163), tumour necrosis factor superfamily member 10 (TNFS10), receptor for advanced glycation endproducts (RAGE), trefoil factor 3 (TFF3), cluster of differentiation 40 ligand (CD40 ligand), interleukin 6 (IL-6), interleukin 8 (IL-8), interleukin 10 (IL-10), interleukin 17A (IL-17A), cardiac troponin I and chemerin using kit LXSAHM-15, respectively. The concentrations of each cytokine were determined using Bio-Rad Bio-Plex 200 system (BioRad, CA, USA).

### Statistical Analysis

A data analysis plan was formulated prospectively and was adhered to. Quantitative data are presented as median and interquartile range (IQR) or mean and standard deviation (SD), as appropriate. Qualitative data are presented by utilising absolute and/or relative frequencies. Data conformity to the normal distribution was analysed by one-sample Kolmogorov-Smirnov testing, and comparisons between patient groups were performed by using one-way ANOVA or the independent samples Kruskal-Wallis H test, as appropriate. For statistically significant differences, post-hoc pairwise comparisons were performed by using Tukey’s honestly significant difference test or Dunn’s test, respectively. Spearman’s rho was used to examine bivariate correlations of quantitative variables. Logarithmic transformations of non-normally distributed variables were used to establish the best linear regression fit during correlation. For the analysis of serial metabolite measurements, the method of summary measures was used^30^. The area under the curve (AUC) for each metabolite was calculated for each participant by using the trapezium rule, as described by Matthews *et al*.^30^. These were standardized by duration of participation in the study, defined by the ultimate time-point of blood sampling for each participant. For the computation of each AUC, any uttermost missing values were omitted, whereas for any middle missing values AUCs were calculated between the immediately adjacent available values. The Kaplan-Meier method was used to compare time-to peak and time-to-minimum metabolite values between groups, and the log-rank test was applied to detect differences; only time-points T0 up to and including T72 were included in the latter analyses.

All statistical tests were based on a two-sided α-value of 0.05. Data were analysed using SPSS^®^ Statistics version 22.0 (IBM Corp., Armonk, NY, USA) and graphs drawn using GraphPad Prism^®^ version 6.0 (GraphPad Software, Inc., La Jolla, CA, USA).

Achieved statistical power was calculated post-hoc using the G-Power algorithm: F tests - ANOVA: Fixed effects, omnibus, one-way^31^. The effect size was computed from measured 3-hydroxykynurenine concentrations in tAP as f = 5.78, with α error probability = 0.05. The noncentrality parameter, λ = 1902.25; Critical F = 3.17; numerator df = 2; denominator df = 54; computed power (1−β error probability) = 1.00.

## Additional Information

**How to cite this article**: Skouras, C. *et al*. Increased levels of 3-hydroxykynurenine parallel disease severity in human acute pancreatitis. *Sci. Rep.* **6**, 33951; doi: 10.1038/srep33951 (2016).

## Supplementary Material

Supplementary Information

## Figures and Tables

**Figure 1:**
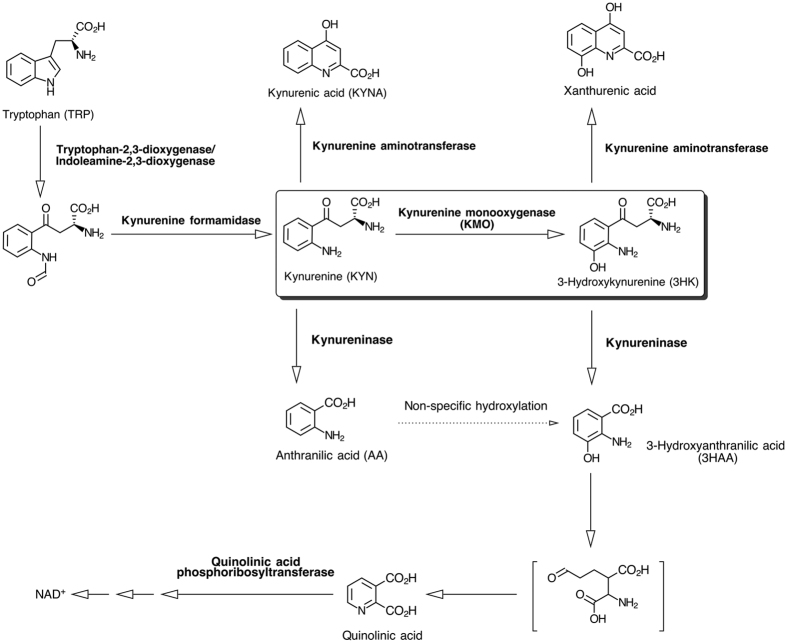
The kynurenine pathway of tryptophan metabolism. The key step of kynurenine conversion to 3-hydroxykynurenine via the action of the gate-keeper enzyme kynurenine 3-monooxygenase (KMO), is highlighted.

**Table 1:** Summary analysis measures^1^ of serial tryptophan and tryptophan metabolite concentrations for the three patient groups of AP severity (T0 to T168).

	Summary Measure	Mild	Moderate	Severe	P-value	HV
Tryptophan (ng/mL)	Minimum values, median (IQR)	16407.2 (13334.9–20463.6)	13993.6 (10714–20941.9)	8820.4 (7295.3–11355.6)	0.071	39763.4
	Mean values, mean (SD)	23840.4 (8970.0)	20346.2 (8144.3)	14421.2 (4317.7)	**0.014**	25215.8 (21675.9–33362.4)
	Time-to-Minimum, median hours (95% C.I.)	12 (3.5–20.5)	12 (0.0–27.7)	12 (3.2–20.8)	0.542	—
	Standardized AUC, mean (SD)	24423.9 (8803.9)	19721.4 (8140.5)	13782.4 (3789.8)	**0.004**	—
Kynurenine (ng/mL)	Peak values, median (IQR)	694.9 (429.4–857.9)	677.8 (408.2–900.8)	742.0 (626.6–1182.8)	0.680	622.3
	Mean values, median (IQR)	520.5 (338.8–708.0)	470.6 (302.1–874.4)	599.4 (547.1–912.4)	0.533	429.2 (353.0–463.1)
	Time-to-Peak, median hours (95% C.I.)	12 (0.0–29.6)	24 (6.3–41.7)	12 (0.0–25.2)	0.288	—
	Standardized AUC, median (IQR)	554.8 (360.3–734.0)	522.7 (370.8–782.5)	634.3 (570.4–840.7)	0.584	—
3-Hydroxykynurenine (ng/mL)	Peak values, median (IQR)	13.5 (8.6–17.8)	20.0 (12.1–46.6)	25.2 (14.3–35.6)	**0.013**	10.5
	Mean values, median (IQR)	10.7 (8.1–13.5)	15.6 (8.6–38.3)	22.3 (11.8–27.0)	**0.039**	8.1 (7.6–9.0)
	Time-to-Peak, median hours (95% C.I.)	12 (6.7–17.3)	46.0* (32.9–59.0)	24 (0.0–49.0)	**0.019**	—
	Standardized AUC, median (IQR)	10.8 (7.4–13.9)	18.2 (11.4–38.1)	21.6 (11.7–28.0)	**0.015**	—
Kynurenic Acid (ng/mL)	Peak values, median (IQR)	211.5 (165.0–404.3)	184.8 (48.2–364.4)	198.4 (182.4–201.6)	0.303	372.1
	Mean values, median (IQR)	194.9 (146.0–364.4)	141.8 (41.8**–**184.8)	186.4 (172.1**–**197.5)	0.104	301.6 (267.7**–**327.5)
	Time-to-Peak value, median hours (95% C.I.)	6 (3.2**–**8.9)	6 (2.7**–**9.3)	3 (0.0**–**7.2)	0.102	—
	Standardized AUC, median (IQR)	296.7 (155.4**–**406.6)	74.1 (34.8**–**203.9)	208.3 (173.8**–**374.3)	0.110	—
3-Hydroxyantranilic Acid (ng/mL)	Peak values, median (IQR)	48.8 (37.3**–**118.6)	48.8 (33.4**–**64.7)	65.0 (46.2**–**394.2)	0.236	54.3
	Mean values, median (IQR)	38.4 (25.9**–**85.3)	34.8 (26.0**–**62.8)	53.6 (41.9**–**215.1)	0.186	37.5 (28.7**–**48.8)
	Time-to-Peak value, median hours (95% C.I.)	6 (0.0**–**12.1)	6 (0.0**–**15.1)	6 (0.0**–**14.8)	0.686	—
	Standardized AUC, median (IQR)	45.7 (28.0**–**98.4)	34.9 (26.8–56.0)	51.2 (44.1–107.5)	0.165	—
3-Hydroxykynurenine/Tryptophan ratio x1000	Peak values, median (IQR)	0.673 (0.417–1.523)	0.878 (0.532–4.515)	1.876 (1.505–3.600)	0.052	0.46
	Minimum values, median (IQR)	0.341 (0.201–0.804)	0.428 (0.283–1.513)	0.872 (0.637–1.420)	**0.005**	0.18
	Mean value, median (IQR)	0.429 (0.321–1.255)	0.645 (0.409–2.902)	1.550 (0.831–1.769)	**0.022**	0.326 (0.248–0.384)
	Time-to-Peak value, median hours (95% C.I.)	12.0 (0.0–26.6)	48 (21.6–74.4)	12 (0.0–24.5)	0.110	—
	Standardized AUC, median (IQR)	0.447 (0.262–0.892)	0.734 (0.506–2.635)	1.667 (0.930–1.841)	**0.003**	—

Standardised AUC were calculated as detailed in the methods section. T168 values were excluded from Time-to-Peak/Minimum analyses. P-values correspond to results from independent samples Kruskal-Wallis H tests or one-way ANOVA comparisons, as appropriate. **HV**: Healthy Volunteers.
