# Supplementary material for: Increased levels of 3-hydroxykynurenine parallel disease severity in human acute pancreatitis
Source: Sci Rep. 2016 Sep 27;6:33951. doi: 10.1038/srep33951 (PMC5037401; doi:10.1038/srep33951)

SUPPLEMENTARY DATA

TITLE

Increased levels of 3-hydroxykynurenine parallel disease severity in human acute pancreatitis

AUTHORS

Christos Skouras[1]*, Xiaozhong Zheng[2], Margaret Binnie[3], Natalie ZM Homer[4], Toby BJ Murray[2], Darren Robertson[5], Lesley Briody[6], Finny Paterson[6], Heather Spence[6], Lisa Derr[6], Alastair J Hayes[2], Andreas Tsoumanis[7], Dawn Lyster[8], Rowan W Parks[1], O James Garden[1], John P Iredale[2], Iain J Uings[9], John Liddle[9], Wayne L Wright[10], George Dukes[11], Scott P Webster[3], Damian J Mole[1,2]

Supplementary Table S1

Demographic characteristics of patients recruited in the IMOFAP study. For the computation of the length of stay, patient re-admission(s) with 10 days from hospital discharge were also taken into account; 'Alcohol use' refers to any alcohol use.

ED: emergency department; **LOS**: length of stay; **ITU**: intensive treatment unit; **HDU**: high-dependency unit; **NA**: not applicable.

	Diagnosis			Modified MODS (Atlanta 2012)		
	Overall	Non-AP	AP	Mild	Moderate	Severe
**No of patients**	79	22 (28%)	57 (72%)	25 (44%)	23 (40%)	9 (16%)
**Gender**						
**Male**	47 (60%)	15	32 (56%)	16	12	4
**Age**						
**Median**	59.6	60.7	57	62.3	54.8	64.8
**IQR**	47.7-73.9	50.1-70.0	47-77.1	46.4-79.0	47.3-67.5	49.4-80.6
**BMI**						
**Mean**	27.2	25.5	27.9	27	28	28
**SD**	6.0	4.7	6.3	5.6	7.4	5.8
**Source of recruitment**						
**ED**	70 (89%)	19	32 (56%)	21	22	8
**Other**	9 (11%)	3	25 (43%)	4	1	1
**LOS**						
**Median**	5	4	5	5	6	7
**IQR**	3-8	2-11	3-8	3-6	3-9	5-13
**Aetiology**						
**Gallstones**	NA	NA	27 (47%)	11	14	2
**Alcohol**	NA	NA	18 (32%)	8	5	5
**Idiopathic**	NA	NA	7 (12%)	2	3	2
**Other**	NA	NA	5 (9%)	4	1	0
**Charlson Index**						
**Median**	2	3	2	2	1	2
**IQR**	1-5	1-5	1-4	1-4	0-3	2-4
**Mortality**						
**In-hospital**	4 (5%)	1	3 (5%)	0	1	2
**30-day**	2 (3%)	0	2 (4%)	0	0	2
**Onset to presentation**						
**Median (hours)**	21.4	46.0	15.3	9.75	23.4	21.3
**IQR (hours)**	8.5-73.3	10.6-135.7	8.3-50.1	5.3-37.8	9.2-75.5	9.2-73.0
**Onset to recruitment**						
**Median (hours)**	33.0	49.5	25.9	18.5	26.7	36.3
**IQR (hours)**	14.2-92.0	18.0-138.5	13.8-70.8	10-46.2	16.6-82.3	17.8-77.8
**Alcohol use**						
**Current**	42 (53%)	10	32 (56%)	15	12	5
**Previous**	8 (10%)	4	4 (7%)	2	2	0
**None**	29 (37%)	8	21 (37%)	8	9	4
**Critical Care admission**						
**ITU**	2 (3%)	0	2 (4%)	0	0	2
**HDU**	5 (6%)	3	2 (4%)	0	0	2
**Overall**	7 (9%)	3	4 (7%)	0	0	4

Supplementary Table S2

Timing of hospital presentation of study participant by day of the week and time of day.

WD: weekday; **WE**: weekend

	Overall		AP		Non-AP	
	N	%	N	%	N	%
**Day of the week**						
**Monday**	9	11	6	11	3	14
**Tuesday**	12	15	8	14	4	18
**Wednesday**	8	10	6	11	2	9
**Thursday**	18	23	14	25	4	18
**Friday**	9	11	7	12	2	9
**Saturday**	9	11	7	1	2	9
**Sunday**	14	18	9	16	5	23
**WD vs. WE**						
**Weekday**	56	71	41	72	15	68
**Weekend**	23	29	16	28	7	32
**Time of day**						
**08:00-15:59**	23	29	13	23	10	46
**16:00-23:59**	32	41	27	47	5	23
**00:00-07:59**	24	30	17	30	7	32
**Overall**	79	100	57	100	22	100

Supplementary Table S3

Summary measures of serial CRP, albumin and cytokine measures, per patient group. Comparisons were performed by utilising one-way ANOVA or Kruskal-Wallis H testing, as appropriate, following normality testing (Kolmogorov-Smirnov). Time-to-Peak and Time-to-Min analyses were performed by applying the Kaplan-Meier method on concentration levels from T0 up to and including T72. *: mean value.

	Summary Measure	Mild	Moderate	Severe	P-value
**Albumin (g/L)**	Peak values, median (IQR)	35 (32-37)	33 (30-37)	26 (24-31)	**0.006**
	Minimum values, median (IQR)	30 (27-32)	27 (23-31)	21 (19-26)	**0.001**
	Mean values, median (IQR)	32.9 (29.6-34.7)	29.8 (26.6-33.4)	22.6 (21.8-28.3)	**0.001**
	Time-to-Min, median hours (95% C.I.)	12.0 (7.8-16.2)	24.0 (16.6-31.4)	21.0 (0.0-47.3)	0.314
	Standardized AUC, median (IQR)	32.5 (29.6-34.0)	28.1 (25.9-31.5)	22.1 (21.3-27.4)	**<0.001**
**C-reactive protein (mg/L)**	Peak values, median (IQR)	97 (32-177)	191 (105-326)	283 (246-391)	**0.001**
	Minimum values, median (IQR)	15 (7-46)	38 (11-96)	103 (88-119)	**0.016**
	Mean values, median (IQR)	53 (22.7-122.2)	125.6 (56.2-190.7)	200.5 (180.6-255.0)	**0.001**
	Time-to-Peak, median hours (95% C.I.)	24.0 (13.7-34.3)	48.0 (40.4-55.6)	24.0 (0.0-76.6)	0.455
	Standardized AUC, median (IQR)	58.9 (26.3-131.2)	148.1 (74.5-243.9)	225.6 (174.9-260.2)	**0.001**
**IL-1 Beta (pg/mL)**	Peak values, median (IQR)	1.1 (0.0-4.5)	4.2 (0.0-7.3)	6.2 (4.2-12.2)	**0.007**
	Minimum values, median (IQR)	0.0 (0.0-2.6)	0.1 (0.0-3.3)	2.6 (0.1-3.5)	0.125
	Mean values, median (IQR)	0.6 (0.0-3.0)	1.9 (0.0-4.9)	4.6 (2.6-6.4)	**0.006**
	Time-to-Peak, median hours (95% C.I.)	3.5* (0.7-6.3)	6.0 (0.0-13.6)	14.7* (0.0-32.0)	0.057
	Standardized AUC, median (IQR)	0.2 (0.0-2.5)	2.6 (0.0-5.1)	4.0 (1.7-6.1)	**0.005**
**IL-6 (pg/mL)**	Peak values, median (IQR)	33.8 (13.4-80.5)	123.4 (44.2-406.1)	904.6 (202.4-1668.8)	**0.001**
	Minimum values, median (IQR)	9.75 (3.2-22.5)	22.5 (8.9-62.6)	40.3 (19.4-66.8)	**0.002**
	Mean values, median (IQR)	19.1 (10.5-56.6)	76.1 (32.8-185.5)	250.3 (116.2-587.7)	**0.001**
	Time-to-Peak, median hours (95% C.I.)	6.0 (0.2-11.8)	12.0 (6.4-17.6)	6.0 (3.6-8.5)	0.345
	Standardized AUC, median (IQR)	20.7 (9.9-53.5)	59.5 (26.8-188.1)	180.8 (83.9-373.2)	**0.003**
**IL-8 (pg/mL)**	Peak values, median (IQR)	16.2 (8.78-29.3)	16.2 (8.6-40.0)	81.8 (15.2-175.2)	0.061
	Minimum values, median (IQR)	4.0 (1.4-7.1)	3.5 (0.6-8.2)	9.4 (7.9-36.7)	**0.022**
	Mean values, median (IQR)	8.9 (5.3-21.1)	9.0 (4.7-22.3)	35.6 (12.3-109.2)	**0.032**
	Time-to-Peak, median hours (95% C.I.)	12.0 (7.3-16.7)	12.0 (6.8-17.2)	6.0 (1.6-10.4)	0.296
	Standardized AUC, median (IQR)	9.4 (5.1-16.6)	8.8 (3.7-20.9)	29.6 (12.2-110.9)	**0.026**
**IL-10 (pg/mL)**	Peak values, median (IQR)	5.4 (2.3-11.4)	9.6 (4.2-18.4)	19.6 (10.1-43.0)	**0.006**
	Minimum values, median (IQR)	2.5 (0.0-4.5)	3.5 (0.0-6.4)	2.4 (0.0-7.0)	0.517
	Mean values, median (IQR)	3.2 (0.5-7.2)	5.6 (1.9-10.5)	14.7 (3.4-28.1)	0.073
	Time-to-Peak, median hours (95% C.I.)	3.0 (0.0-6.7)	12.0 (4.6-19.4)	8.0* (0.0-19.1)	0.369
	Standardized AUC, median (IQR)	4.1 (0.29-6.97)	4.6 (2.0-7.3)	9.2 (3.3-18.2)	0.209
**IL-17 (pg/mL)**	Peak values, median (IQR)	1.4 (0.0-3.8)	0.1 (0.0-2.2)	7.4 (3.0-12.8)	0.087
	Minimum values, median (IQR)	0.0 (0.0-0.0)	0.0 (0.0-0.0)	2.1 (0.9-3.5)	**<0.001**
	Mean values, median (IQR)	0.4 (0.0-1.0)	0.0 (0.0-0.9)	5.2 (1.6-8.2)	0.061
	Time-to-Peak, median hours (95% C.I.)	6.0 (0.4-11.6)	12.0 (5.6-18.4)	3.0 (0.0-6.9)	0.914
	Standardized AUC, median (IQR)	0.4 (0.0-1.0)	0.1 (0.0-1.2)	3.6 (1.4-7.6)	0.069

**CA 15-3 (pg/mL)**	Peak values, median (IQR)	20.2 (13.8-31.0)	15.0 (11.6-20.8)	18.5 (17.5-30.5)	0.083
	Minimum values, median (IQR)	12.4 (8.8-16.8)	8.0 (5.3-12.0)	11.0 (8.5-23.0)	0.072
	Mean values, median (IQR)	17.6 (13.0-21.8)	11.7 (6.8-16.3)	14.6 (11.8-26.7)	0.079
	Time-to-Min, median hours (95% C.I.)	6.0 (4.0-8.0)	24.0 (18.7-29.3)	12.0 (0.0-38.3)	**0.008**
	Standardized AUC, median (IQR)	16.7 (12.3-22.5)	10.9 (6.4-14.3)	15.2 (11.2-27.3)	**0.035**
**Insulin (pg/mL)**	Peak values, median (IQR)	1337.2 (953.7-1758.1)	1212.7 (600.6-2222.6)	764.2 (680.5-1630.3)	0.244
	Minimum values, median (IQR)	343.6 (230.1-641.6)	375.3 (198.9-619.3)	274.5 (197.3-330.7)	0.384
	Mean values, median (IQR)	742.1 (573.2-985.1)	780.2 (368.5-1350.6)	538.5 (506.3-650.3)	0.234
	Time-to-Min, median hours (95% C.I.)	NA	NA	NA	NA
	Standardized AUC, median (IQR)	760.5 (588.6-1123.7)	744.4 (478.0-1290.8)	440.0 (349.8-566.3)	**0.042**
**Insulin C-Peptide (pg/mL)**	Peak values, median (IQR)	3138.0 (2457.3-3817.9)	1898.8 (1135.7-3032.3)	1590.3 (863.4-4067.4)	0.205
	Minimum values, median (IQR)	976.0 (427.0-1636.4)	559.4 (119.1-1278.9)	421.2 (236.2-1294.4)	0.350
	Mean values, median (IQR)	1902.8 (1205.5-2725.3)	1193.2 (625.9-2040.3)	660.4 (515.5-2244.2)	0.311
	Time-to-Peak, median hours (95% C.I.)	24.0 (12.5-35.5)	24.0 (0.0-50.4)	6.0 (0.0-14.8)	0.548
	Standardized AUC, median (IQR)	1955.7 (1371.3-2715.2)	1320.5 (874.0-2096.0)	833.8 (501.7-2688.2)	0.324
**CD40 ligand (ng/mL)**	Peak values, median (IQR)	1.6 (0.9-2.6)	2.4 (1.5-3.9)	2.9 (1.2-3.1)	0.162
	Minimum values, median (IQR)	0.5 (0.4-0.7)	0.7 (0.4-1.1)	0.6 (0.3-0.7)	0.153
	Mean values, median (IQR)	0.9 (0.6-1.5)	1.3 (0.9-2.4)	1.1 (0.8-1.7)	0.092
	Time-to-Peak, median hours (95% C.I.)	6.0 (0.5-11.5)	3.0 (0.0-13.7)	12.0 (3.7-20.3)	0.947
	Standardized AUC, median (IQR)	0.8 (0.6-1.7)	1.5 (0.7-2.1)	1.0 (0.7-1.8)	0.199
**Chemerin (ng/mL)**	Peak values, median (IQR)	11.6 (8.9-13.6)	14.5 (11.8-17.0)	12.4 (10.3-17.0)	0.081
	Minimum values, median (IQR)	8.5 (6.7-10.7)	8.6 (6.6-12.7)	8.1 (5.7-9.2)	0.588
	Mean values, median (IQR)	9.9 (7.6-11.3)	10.7 (7.8-14.6)	9.6 (8.0-10.6)	0.345
	Time-to-Peak, median hours (95% C.I.)	24.0 (15.8-32.2)	48.0 (32.8-63.2)	48.0 (14.7-81.3)	0.293
	Standardized AUC, median (IQR)	9.9 (7.6-11.9)	11.3 (9.7-14.1)	9.5 (8.1-11.8)	0.127
**TFF3 (ng/mL)**	Peak values, median (IQR)	3.7 (3.1-4.6)	3.7 (3.1-5.6)	7.0 (5.4-8.4)	**0.008**
	Minimum values, median (IQR)	3.0 (2.4-3.9)	3.3 (2.4-4.2)	4.5 (4.0-6.4)	**0.025**
	Mean values, median (IQR)	3.3 (2.7-4.2)	3.5 (2.6-4.7)	5.4 (4.8-7.6)	**0.011**
	Time-to-Peak, median hours (95% C.I.)	6.0 (1.1-10.9)	12.0 (0.4-23.6)	6.0 (1.7-10.3)	0.719
	Standardized AUC, median (IQR)	3.33 (2.72-4.1)	3.5 (2.8-4.9)	5.2 (4.9-7.5)	**0.013**
**RAGE (ng/mL)**	Peak values, median (IQR)	2.3 (1.6-3.4)	2.1 (1.5-3.3)	2.8 (2.3-4.2)	0.269
	Minimum values, median (IQR)	1.7 (1.2-2.2)	1.5 (1.0-2.1)	1.9 (1.8-2.0)	0.374
	Mean values, median (IQR)	1.9 (1.3-2.8)	1.7 (1.3-2.6)	2.2 (2.1-3.8)	0.228
	Time-to-Peak, median hours (95% C.I.)	6.0 (3.6-8.4)	24.0 (15.6-32.5)	12.0 (3.7-20.3)	0.455
	Standardized AUC, median (IQR)	1.9 (1.4-2.7)	1.9 (1.6-2.6)	2.1 (2.0-3.3)	0.370
**CD163 (ng/mL)**	Peak values, median (IQR)	872.3 (585.3-1271.3)	1058.6 (827.2-1341.1)	1996.4 (1196.6-2640.0)	**0.017**
	Minimum values, median (IQR)	639.8 (449.7-996.6)	683.7 (543.95-897.8)	834.9 (699.8-1046.0)	0.480
	Mean values, median (IQR)	778.0 (531.9-1137.2)	861.9 (723.4-1171.5)	1636.0 (1003.7-1846.8)	**0.031**
	Time-to-Peak, median hours (95% C.I.)	3.0 (0.0-6.6)	3.0 (1.4-4.6)	6.0 (3.2-8.8)	0.112
	Standardized AUC, median (IQR)	743.74 (493.0-1143.4)	833.4 (664.4-1052.9)	1417.9 (952.8-1812.5)	**0.028**
**IFN-γ (pg/mL)**	Peak values, median (IQR)	0.0 (0.0-0.0)	0.0 (0.0-0.5)	0.0 (0.0-1.2)	0.362
	Minimum values, median (IQR)	0.0 (0.0-0.0)	0.0 (0.0-0.0)	0.0 (0.0-0.0)	0.162
	Mean values, median (IQR)	0.0 (0.0-0.0)	0.0 (0.0-0.2)	0.0 (0.0-0.3)	0.295
	Time-to-Peak, median hours (95% C.I.)	3.0 (0.0-12.6)	12.0 (0.3-23.7)	36.0* (17.6-43.6)	0.990
	Standardized AUC, median (IQR)	0.0 (0.0-0.0)	0.0 (0.0-0.2)	0.0 (0.0-0.6)	0.177
**TNF-α (pg/mL)**	Peak values, median (IQR)	1.3 (0.0-8.5)	3.1 (0.0-6.1)	9.9 (3.5-34.5)	**0.031**
	Minimum values, median (IQR)	0.0 (0.0-0.6)	0.0 (0.0-1.8)	1.3 (0.1-3.6)	0.079
	Mean values, median (IQR)	0.6 (0.0-3.8)	1.2 (0.0-3.2)	7.6 (2.6-15.5)	**0.010**
	Time-to-Min, median hours (95% C.I.)	3.0 (0.0-6.9)	6.0 (0.0-29.8)	12.0 (0.0-29.5)	0.561
	Standardized AUC, median (IQR)	0.4 (0.0-5.1)	1.1 (0.0-2.9)	7.2 (2.5-8.4)	**0.017**

**B7-H1 (pg/mL)**	Peak values, median (IQR)	0.0 (0.0-85.4)	0.0 (0.0-0.0)	0.0 (0.0-0.0)	0.160
	Minimum values, median (IQR)	0.0 (0.0-0.0)	0.0 (0.0-0.0)	0.0 (0.0-0.0)	0.550
	Mean values, median (IQR)	0.0 (0.0-14.2)	0.0 (0.0-0.0)	0.0 (0.0-0.0)	0.184
	Time-to-Peak, median hours (95% C.I.)	NA	NA	NA	NA
	Standardized AUC, median (IQR)	0.0 (0.0-3.5)	0.0 (0.0-0.0)	0.0 (0.0-0.0)	0.370
**TRAIL-TNFS10 (pg/mL)**	Peak values, median (IQR)	30.4 (13.7-71.3)	20.26 (11.5-37.6)	31.5 (0.0-34.5)	0.382
	Minimum values, median (IQR)	10.8 (1.0-25.8)	10.1 (0.0-22.6)	14.1 (0.0-17.3)	0.639
	Mean values, median (IQR)	22.9 (7.9-36.9)	15.0 (4.3-33.9)	18.7 (0.0-25.9)	0.368
	Time-to-Peak, median hours (95% C.I.)	6.0 (3.2-8.8)	12.0 (7.2-16.8)	3.0 (0.0-17.4)	0.707
	Standardized AUC, median (IQR)	20.4 (7.6-45.2)	13.1 (5.1-30.7)	18.9 (0.0-24.4)	0.292
**CXCL12 (pg/mL)**	Peak values, median (IQR)	0.0 (0.0-0.0)	0.0 (0.0-0.0)	0.0 (0.0-11.76)	**0.045**
	Minimum values, median (IQR)	0.0 (0.0-0.0)	0.0 (0.0-0.0)	0.0 (0.0-0.0)	0.335
	Mean values, median (IQR)	0.0 (0.0-0.0)	0.0 (0.0-0.0)	0.0 (0.0-4.1)	**0.040**
	Time-to-Peak, median hours (95% C.I.)	NA	NA	NA	NA
	Standardized AUC, median (IQR)	0.0 (0.0-0.0)	0.0 (0.0-0.0)	0.0 (0.0-5.0)	**0.023**
**Cardiac Troponin I/c TNI (pg/mL)**	Peak values, median (IQR)	9.4 (0.0-18.3)	13.9 (0.0-38.3)	14.3 (7.6-26.1)	0.516
	Minimum values, median (IQR)	0.0 (0.0-0.0)	0.0 (0.0-0.0)	0.0 (0.0-0.0)	0.380
	Mean values, median (IQR)	1.9 (0.0-9.2)	4.64 (0.0-11.0)	4.8 (3.1-8.5)	0.868
	Time-to-Peak, median hours (95% C.I.)	6.0 (3.8-8.2)	48.0 (22.9-73.1)	3.0 (0.0-6.5)	**0.010**
	Standardized AUC, median (IQR)	1.5 (0.0-10.3)	5.4 (0.0-10.7)	4.3 (2.6-12.6)	0.772

Supplementary Table S4

Summary of P-values from post-hoc pairwise comparisons of significantly different standardized AUC of metabolites between patient groups, adjusted according to Dunn's test. Significant P-values are highlighted in bold. (*: Tukey honestly significant difference test).

Metabolite	Pairwise Standardized AUC Comparison (Adjusted P-values)		
	Mild vs. Moderate	Mild vs. Severe	Moderate vs. Severe
**Tryptophan***	0.128	**0.003**	0.156
**3-Hydroxykynurenine**	**0.040**	0.064	1.000
**3-HK/TRP x1000**	0.057	**0.004**	0.493
**Albumin**	**0.028**	**<0.001**	0.171
**C-reactive protein**	**0.031**	**0.001**	0.367
**IL-1 Beta**	0.144	**0.005**	0.311
**IL-6**	0.090	**0.003**	0.304
**IL-8**	1.000	**0.043**	**0.032**
**CA 15-3**	0.058	1.000	0.158
**Insulin**	1.000	**0.044**	0.086
**TFF3**	1.000	**0.014**	**0.028**
**CD163**	1.000	**0.025**	0.083
**TNF-α**	1.000	**0.026**	**0.024**
**CXCL12**	1.000	**0.031**	**0.035**

Supplementary Figure S5

Schematic representation of the IMOFAP scheduler mode of function. The scheduler populates the sequence of time-points for each consecutive participant in a digital calendar, as demonstrated below (orange colour: participant 1, blue colour: participant 2).

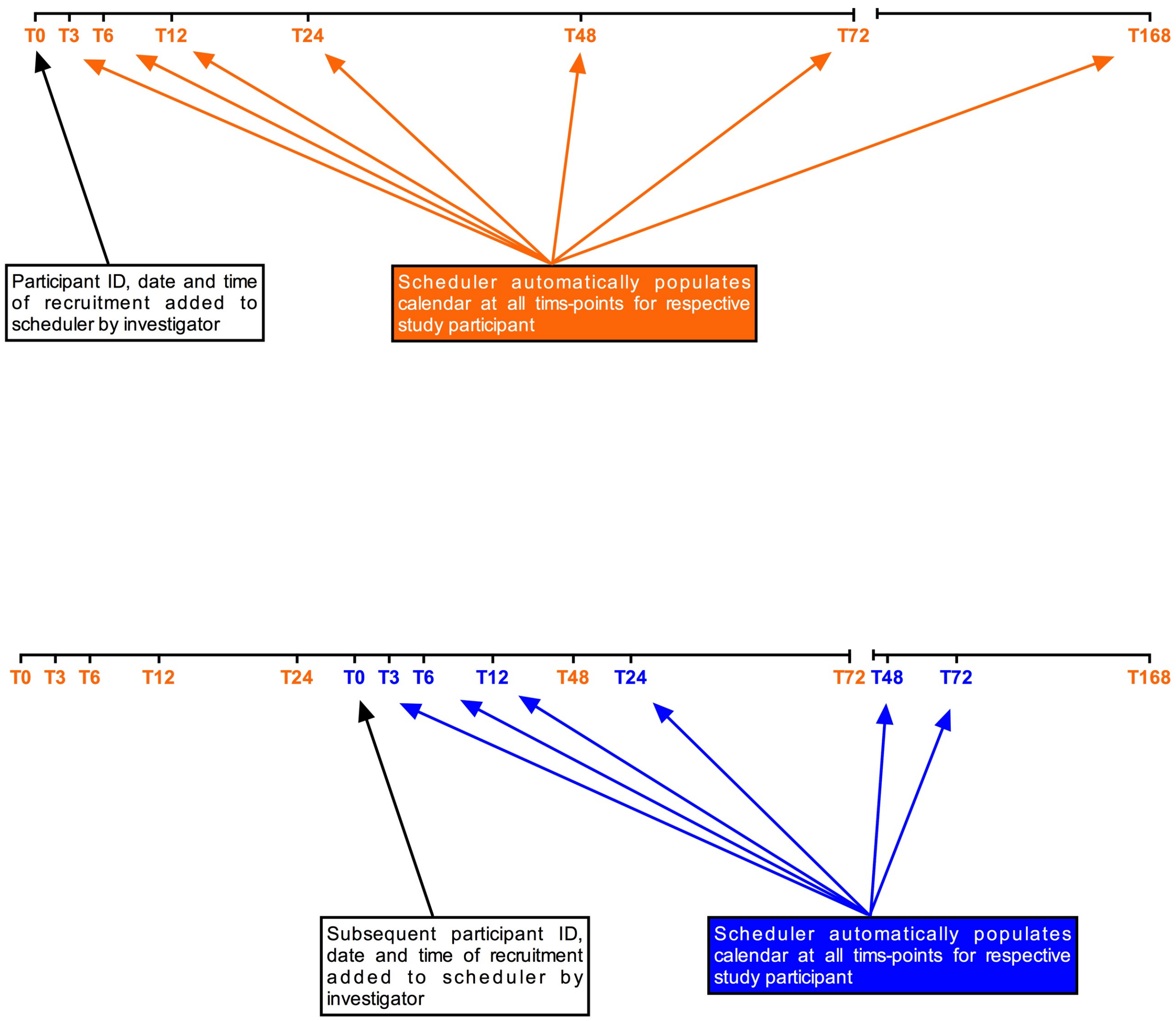

Supplement: Supplementary Information [file srep33951-s1.pdf]
